# Gigapixel surface imaging of radical prostatectomy specimens for comprehensive detection of cancer-positive surgical margins using structured illumination microscopy

**DOI:** 10.1038/srep27419

**Published:** 2016-06-03

**Authors:** Mei Wang, David B. Tulman, Andrew B. Sholl, Hillary Z. Kimbrell, Sree H. Mandava, Katherine N. Elfer, Samuel Luethy, Michael M. Maddox, Weil Lai, Benjamin R. Lee, J. Quincy Brown

**Affiliations:** 1 Department of Biomedical Engineering, Tulane University, New Orleans, LA 70118, USA; 2 Bioinnovation Program, Tulane University, New Orleans, LA 70118, USA; 3 Department of Pathology and Laboratory Medicine, Tulane University School of Medicine, New Orleans, LA 70112, USA; 4 Department of Urology, Tulane University School of Medicine, New Orleans, LA 70112, USA.

## Abstract

Achieving cancer-free surgical margins in oncologic surgery is critical to reduce the need for additional adjuvant treatments and minimize tumor recurrence; however, there is a delicate balance between completeness of tumor removal and preservation of adjacent tissues critical for normal post-operative function. We sought to establish the feasibility of video-rate structured illumination microscopy (VR-SIM) of the intact removed tumor surface as a practical and non-destructive alternative to intra-operative frozen section pathology, using prostate cancer as an initial target. We present the first images of the intact human prostate surface obtained with pathologically-relevant contrast and subcellular detail, obtained in 24 radical prostatectomy specimens immediately after excision. We demonstrate that it is feasible to routinely image the full prostate circumference, generating gigapixel panorama images of the surface that are readily interpreted by pathologists. VR-SIM confirmed detection of positive surgical margins in 3 out of 4 prostates with pathology-confirmed adenocarcinoma at the circumferential surgical margin, and furthermore detected extensive residual cancer at the circumferential margin in a case post-operatively classified by histopathology as having negative surgical margins. Our results suggest that the increased surface coverage of VR-SIM could also provide added value for detection and characterization of positive surgical margins over traditional histopathology.

The first intra-operative frozen section analysis to confirm a diagnosis of cancer during surgery was performed in 1895^1^. It is therefore surprising that 120 years later, this technology remains the clinical reference standard for intra-operative determination of completeness of oncologic surgical excision. Although it is the most long-standing intra-operative pathology method, it still represents a sub-optimal sampling method, and as a result has not eliminated the problem of incomplete tumor resections.

The consequences of an incomplete surgical tumor resection include the need for additional painful and harmful salvage treatments, an increase in overall treatment costs, and most importantly, an increased chance of tumor recurrence and cancer-related morbidity and mortality. Surgical removal of the tumor is the frontline curative strategy for non-metastatic cancers of many solid organs, and the success of the strategy depends on complete removal of the tumor during the primary operation – i.e., achieving negative surgical margins (NSM), or the absence of residual disease at the removed tissue surface. However, positive surgical margins (PSM), or tumor extending to the surface of the resection specimen, remain a significant problem in organ sites with large resection specimens such as the breast and prostate^2^,^3^,^4^. Specifically for prostate cancer, 11–38% of radical prostatectomy (RP) operations result in a PSM regardless of clinical stage^3^ and can exceed 50% in the highest stage (pT3 and pT4) tumors^5^. PSM’s are associated with increased biochemical and local tumor recurrence and are generally accepted as a poor independent prognostic indicator^3^,^6^,^7^. The only currently available solution is intra-operative targeted frozen section analysis (targeted FSA), in which small focal shavings of the tissue surface considered suspicious by the surgeon are sent for analysis, the result of which is used to attempt to correct a PSM. However, due to the labor- and time-intensiveness of FSA, this approach is limited to analysis of small areas. This is further compounded by the fact that there are no reliable and reproducible clinical or visual indicators for accurately identifying suspicious areas on the prostate surface for FSA sampling. These factors have likely contributed to the high false negative rate (low sensitivity) of targeted FSA in prostate cancer surgery^3^,^8^,^9^.

Consequently, the current gold-standard for identification of prostate PSMs is not FSA but rather post-operative formalin-fixed permanent histopathology; however, because the latter is performed after the surgery it is not helpful for identifying and correcting a PSM intra-operatively. Furthermore, due to the bulky geometry, irregular contours, and large size of the prostate, it is not possible to obtain a circumferential section of the entire prostate surface even with standard histologic processing. (To put the difficulty of this task in context, it is useful to imagine the difficulties one would have in peeling an apple at 4 μm thickness uniformly over its entire surface.) Since taking a circumferential section of the entire prostate is not feasible using standard pathology techniques, the primary impediment to intra-operative pathology is the time required to serially section large volumes of the tissue into thin cross-sections that can then be mounted on microscope slides, stained, and observed using standard light-transmission microscopy.

*Ex vivo* microscopy is a set of emerging optical techniques that aim to address this limitation by moving the acquisition of the diagnostic histologic image from the microscope slide to the fresh specimen itself, using advanced optical techniques to eliminate the intensive cutting steps. Techniques applied to date have included depth-sensitive tomography techniques such as optical coherence tomography^10^,^11^,^12^,^13^,^14^ and photoacoustic tomography^15^, as well as optical sectioning techniques such as reflectance and fluorescence scanning confocal microscopy and label-free nonlinear microscopies^16^,^17^,^18^,^19^,^20^,^21^,^22^,^23^,^24^,^25^,^26^,^27^,^28^,^29^. These techniques have shown promising results for imaging of smaller specimens such as skin cancer resections^17^,^21^, gross pathology sections of larger resection specimens^16^,^17^,^25^,^27^, and core needle biopsies^30^. However, subcellular resolution images of fully intact tumor resection specimen surfaces have yet to be delivered, presumably due to the throughput challenges associated with sequential beam-scanning approaches. The throughput limitation for beam scanning approaches is the pixel dwell time, which is the time required at each pixel to collect enough fluorescent photons to obtain good signal-to-noise ratios, usually on the order of microseconds per pixel. However, the fundamental challenge for imaging large tumor resection specimens at high resolution with *ex vivo* microscopy is that the number of measurements (i.e. pixels) acquired within a given amount of intra-operative time must be maximized to achieve the opposing goals of high spatial resolution and high area coverage; therefore, very high throughput methods are needed.

An alternative technology that our group has been advancing for *ex vivo* microscopy is optical-sectioning fluorescence structured illumination microscopy^31^,^32^,^33^. Structured illumination microscopy (SIM) is a light-efficient wide-field optical sectioning technique^34^,^35^ that has the advantage of parallel pixel acquisition, such that the overall pixel-sampling frequency scales most strongly with the pixel count of the detector rather than the single-pixel exposure time or dwell time. The frame rate and pixel density achieved in our system would require an equivalent beam-scanning pixel dwell time of 7 ns, which represents a fundamental advance in speed for fluorescence *ex vivo* microscopy compared to beam-scanning approaches. Importantly, SIM provides this speed advantage while simultaneously providing the reduced background signal and attendant increase in contrast and effective resolution given by optical sectioning methods. We recently completed a validation study of video-rate SIM (VR-SIM) on large core prostate biopsies against gold-standard H&E, in which the diagnostic accuracy on pathologist review resulted in an area under the ROC curve of 0.82–0.88, even in the presence of limited prostate adenocarcinoma content (average 13.7% tumor content per malignant biopsy)^33^. The speed and diagnostic accuracy of VR-SIM thus position it as a potentially useful tool for prostate cancer detection and diagnosis in the surgical pathology setting.

In this work we sought to investigate the potential of VR-SIM as a practical and useful tool for intra-operative microscopy of fully intact human prostates, with the goal of identifying microscopic prostate adenocarcinoma at the surgical margin. In this report we demonstrate the first gigapixel “histologic landscape” *ex vivo* microscopy images of entire radical prostatectomy (RP) resection margin surfaces taken immediately after surgical excision, without cutting or any physical manipulation of the surgical margin. This imaging approach “peels the apple” by collecting a thin optical section of the prostatic circumferential surface, rather than a thin physical section. Such an approach has the advantages of capturing the tissue surface plane of interest in its entirety, increasing the chances that tumor involvement will be captured; reducing the amount of non-surface-related tissue information that must be examined by the pathologist for margin analysis; and being non-destructive, therefore not compromising historical standard-of-care analyses. We show that even at current non-optimized microscope stage automation speeds it is feasible to routinely image entire prostate resection surfaces in ≤1 hour at subcellular resolution, including specimen handling and staining times. Importantly, our findings suggest that intra-operative use of VR-SIM may be useful not only in confirming the presence of a PSM in a more timely manner, enabling adjustment of the surgery, but in fact it may enable detection of clinically-significant positive surgical margins missed by permanent histopathology, which would have clear implications for patient management. Overall these results position *ex vivo* microscopy with structured illumination microscopy as a practical, routine, non-destructive intra-operative method for robust circumferential tumor margin evaluation.

## Results

### Video-rate structured illumination microscopy (VR-SIM)

**Figure 1:**
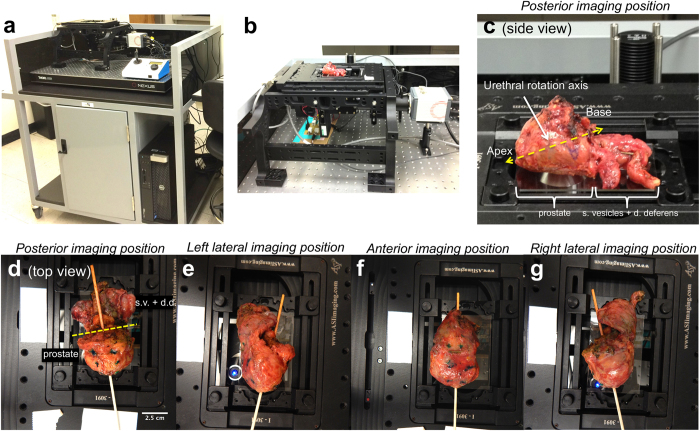
VR-SIM imaging system and prostate circumferential surface imaging procedure. (**a**) The VR-SIM system is mounted on a movable cart. (**b**) Close-up of the VR-SIM imaging system with a prostate on the microscope stage. The imaging objective is below the prostate in an epi-illumination configuration. (**c**) Close-up (side view) of the posterior imaging position of the prostate. The posterior surface is contacting the slide; the imaging objective is seen below the prostate. The prostate is rotated about the urethral axis (yellow dashed line) to enable imaging of the prostate circumference. The prostate and the seminal vesicles/ductus deferens are indicated in the photograph. (**d**–**g**) Top views of the circumferential imaging positions (posterior, left lateral, anterior, right lateral). A wooden dowel rod is inserted through the urethra to demonstrate the rotation axis between imaging positions. In (**d**), the plane between the prostate and the attached seminal vesicles (s.v.) and ductus deferens (d.d.) is indicated by a dashed yellow line.

We have previously reported the development of an automated optical sectioning microscopy scanner for large tissues stained with acridine orange using a video-rate implementation of structured illumination microscopy (Fig. 1a,b)^32^. The microscope setup and imaging method is described in detail in the Methods. Briefly, the system consists of a custom structured illumination module attached to a commercial automated modular epi-fluorescence microscope platform, with custom control and acquisition software. Images of large areas are achieved by stepping the prostate over the objective lens and assembling mosaics of the resulting frames. In this work, each individual SIM frame was collected in 30 ms thereby achieving a pixel-sampling frequency of 139.8 megapixels/sec. Accounting for the stage-scanning time (~200 ms between successive image frames), the real image acquisition throughput in this study was 18 megapixels/sec, with a Nyquist-limited lateral resolution of 1.3 μm.

### Circumferential imaging of radical prostatectomy specimens

We imaged the circumference of fully-intact prostates from 24 patients providing informed consent under an IRB-approved protocol from September 2014 to October 2015 who were undergoing robotic-assisted laparoscopic radical prostatectomy for pathologically-confirmed prostate cancer at Tulane University Medical Center. The prostates were transported to a nearby imaging suite immediately after removal from the patient, and the imaging team imaged the prostate for approximately one hour before forwarding the specimen for standard-of-care histopathology processing. The first 5 prostates were used to optimize specimen handling and staining protocols and image acquisition parameters, and were not included in the final analysis. For the following 19 patients (with the exception of one described below), using the protocol established in the first 5 patients, we imaged 4 circumferential surfaces of the prostate (right lateral, left lateral, posterior, and anterior) in their entirety within the one-hour timeframe. For imaging, the stained prostate was positioned on a 50 × 75 × 1 mm glass slide on the VR-SIM stage above the imaging objective (Fig. 1c). Multiple images of the prostate circumference were obtained by manually rotating and re-positioning the stained prostate and collecting mosaic images of the surface contacting the glass slide (Fig. 1d–g). Supplementary Table S1 lists the pathologic tumor stage, tumor Gleason grade, number of histology H&E slides analyzed post-operatively, and the final post-operative surgical margin status by location (left posterior and anterior quadrants, right posterior and anterior quadrants, base, and apex) for each of the patients retained for analysis. In Case 8, an additional 5^th^ surface was imaged (the prostate base). In Case 10 an isolated hardware malfunction occurred and only one margin surface was imaged in the allotted timeframe. Of the 19 specimens imaged and retained for analysis, 8 had a PSM on permanent histopathology. For four of these specimens (Cases 12, 17, 20, and 23, all stage pT2c), the PSM was found on the apical surface of the prostate only, which was not directly imaged with VR-SIM in this study.

**Figure 2:**
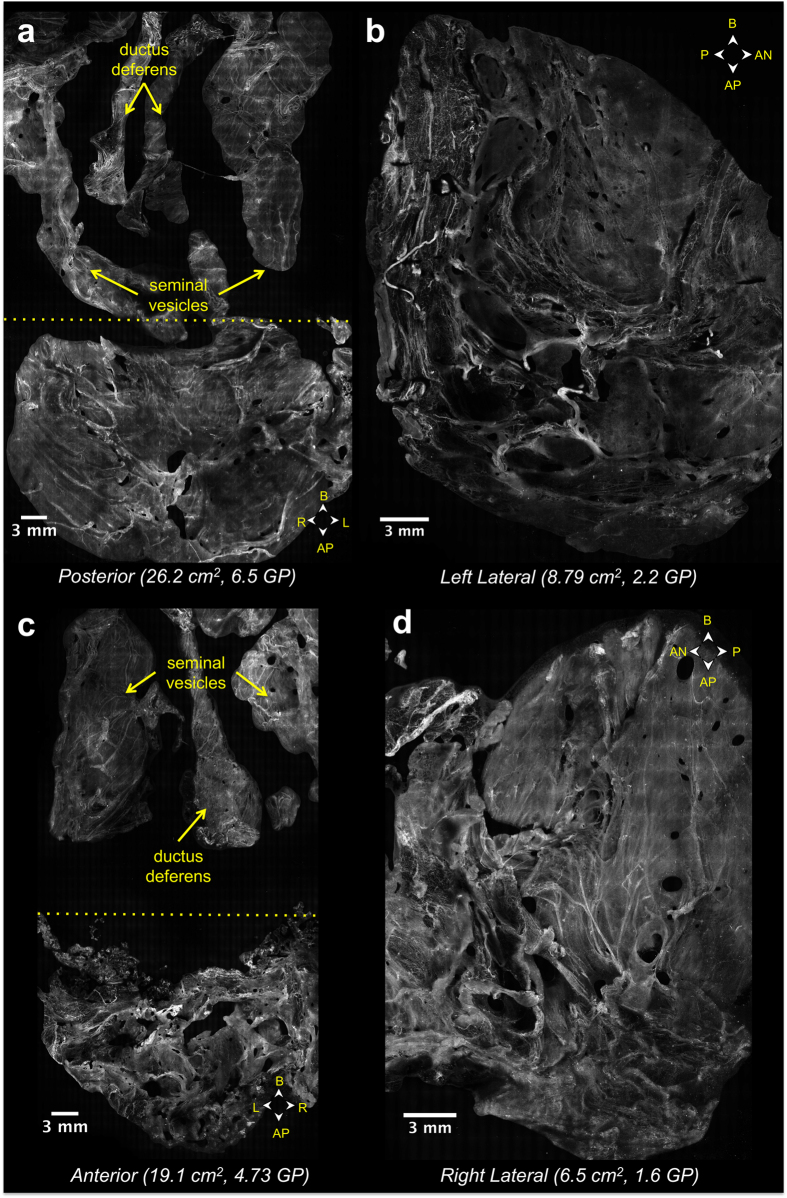
Circumferential VR-SIM surface images of the prostate of Case 6. (**a**) Posterior prostate surface, with seminal vesicles and ductus deferens situated above the dashed yellow line, (**b**) left lateral prostate surface, (**c**) anterior prostate surface, with portions of the seminal vesicles and ductus deferens visible above the dashed yellow line and (**d**) right lateral prostate surface. The image area and image size in gigapixels (GP) is provided for each image, and totals 60.5 cm^2^ and 15 GP in all. The orientation of each image with respect to the prostate apex (AP), base (B), anterior surface (AN), posterior surface (P), left lateral surface (L), and right lateral surface (R) is indicated by the direction arrows in each image.

Figure 2 contains VR-SIM mosaic images of the four circumferential surface aspects of the RP specimen of Case 6, with compass arrows in each image indicating the anatomical orientation of the imaged surfaces. Figure 2a,c contain VR-SIM images of the opposing anterior and posterior surfaces of the prostate; these images also contain portions of the seminal vesicles and ductus deferens, which originate at the posterior prostate base and extend upward from the prostate in these images (demarcated by the dashed yellow line). Figure 2b,d contain VR-SIM images of the opposing left and right lateral surfaces of the same prostate (the seminal vesicles and ductus deferens are not imaged in these orientations). The total image area for this prostate was 60.5 cm^2^, corresponding to 15 gigapixels of image data at 0.65 μm/pixel (Nyquist-limited lateral resolution of 1.3 μm).

**Figure 3:**
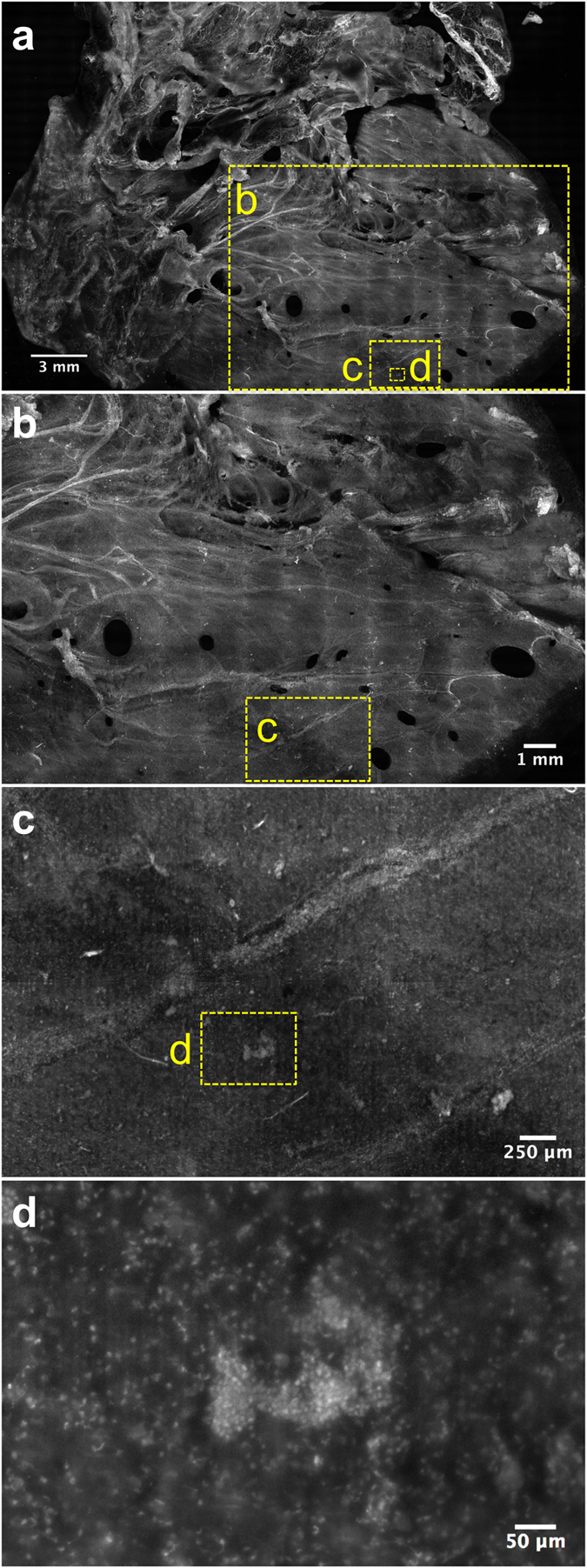
Multi-scale visualization of prostate surface images from the macro-scale to the micro-scale. (**a**) VR-SIM image of the 6.5 cm^2^ right lateral surface of Case 6, (**b**) Zoom of the large dashed yellow box area indicated in (**a**). Features such as the neurovascular bundles (bright spindly features) and smooth prostatic pseudocapsule/fascia are apparent. (**c**) Zoom of the dashed yellow box in (**b**). The image is marked by a single neurovascular bundle extending across the field of view. A bright feature (**d**) is enclosed by a dashed yellow box, corresponding to a single prostate gland. Individual rounded nuclei are clearly resolved.

A VR-SIM image from a single surface plane usually exceeds several gigabytes in file size; therefore, it is most practical to convert the images to multi-resolution tiled pyramidal TIFF or BigTIFF files, which can then be rapidly viewed using multi-resolution ‘zoom and pan’ viewers such as ImageScope or web-based viewers such as Gigapan or IIPImage. Figure 3 depicts a series of zooms into the VR-SIM image of the left lateral surface image from Fig. 2b, demonstrating the multi-scale nature of these gigapixel images, spanning 5 orders of length magnitude in a single image, from a macroscopic view of the whole organ surface (centimeter-scale, Fig. 3a) to the visualization of individual cell nuclei (micrometer-scale, Fig. 3d). The multi-scale nature of these gigapixel images allows the reviewing pathologist to identify areas of interest based on known prostate anatomy or regions of morphological interest, and to dynamically zoom into the image to observe the tissue at the cellular level for diagnostic confirmation (Supplementary Video 1).

### Construction of an image atlas for interpretation of VR-SIM images of the prostate surface

Current standard-of-care histopathology protocols to identify benign or cancerous glands at the prostate surface require that the prostate first be cut grossly into quadrants along the urethral axis, each of which are then ‘bread-loafed’ into ~3 mm-thick gross cross-sections along the urethral axis of the prostate. From each of these 3 mm-thick slices, a single 4 μm-thick tissue section is obtained for H&E staining and eventual analysis (Supplementary Fig. S1). Since VR-SIM imaging is directly applied to the prostate surface to obtain a virtual circumferential section, the current clinical tissue processing standards do not allow for an identical histology section of the *en face* prostate surface to be obtained for co-registration with the VR-SIM images. Therefore, we developed a clinical image atlas of VR-SIM images against histopathology to aid in interpretation of the prostate surface image features.

To assemble the atlas, VR-SIM images of both normal and malignant prostate glandular parenchyma, as well as of prostate surface features, were needed along with corresponding histopathology sections. In previous work, we conducted an imaging study of prostate large core biopsies to determine the accuracy with which pathologists can distinguish benign prostate from malignant prostate^33^. However, the previous study on core biopsies did not contain samples of the undisturbed prostate surface. Therefore, we assembled images of prostate surface features to include in the atlas from two additional tissue sources: 1) a VR-SIM image of a fresh cadaveric prostate surface with a corresponding *en face* histological section of the surface, and 2) radical prostatectomy specimens with cross-sectional histological sections sampled from areas located approximate to features found in VR-SIM surface images. For (1), we simulated an intra-prostatic incision on a fresh cadaveric prostate under an IRB-approved protocol, followed by collection of an *en face* section of the prostate surface corresponding to the VR-SIM image. Specifically, a quadrant of the prostate was obtained and an oblique section of the upper half of the prostate surface was then removed with a razor blade to simulate an incision through the prostate capsule. The outer surface of the prostate, now including regions of both normal prostate capsule and surgically-exposed glandular parenchyma, was stained with acridine orange and imaged using VR-SIM. The tissue was subsequently fixed in formalin and an *en face* section of the prostate surface was obtained and processed for H&E. For (2), prominent features on the VR-SIM radical prostatectomy surface images were correlated from clinical standard cross-sectional H&E sections taken from blocks corresponding to the approximate location of the feature of interest.

**Figure 4:**
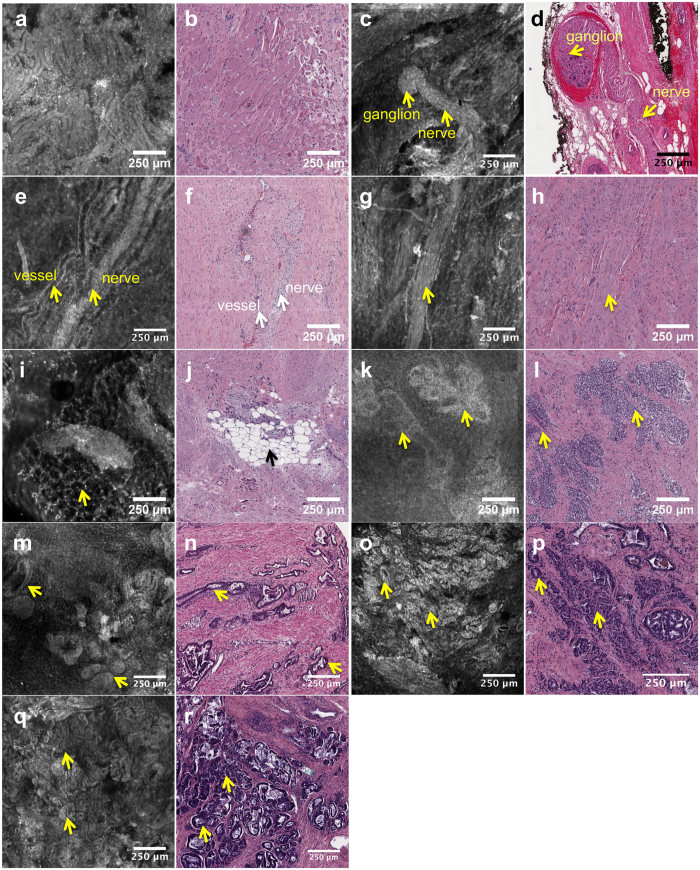
Clinical image atlas of VR-SIM versus histopathology images of benign and malignant prostate structures. Arrows indicate corresponding features between VR-SIM and H&E-stained histology slides. (**a,b**) normal skeletal muscle, (**c,d**) nerves with ganglia, (**e,f**) neurovascular bundle, (**g,h**) smooth muscle bundle, (**i,j**) adipose tissue, (**k,l**) normal prostate glands with ‘cauliflower’ appearance, (**m,n**) benign prostate glands with elongated and rounded appearances, (**o–r**) examples of prostate adenocarcinoma, acinar type, Gleason grade 3 + 4 = 7.

Now including images from prostatic surface features as well as benign and malignant prostatic glandular parenchyma, the atlas includes all features that, based on known prostate microanatomy, are reasonably expected to be observed at the prostate surface. Figure 4 contains sample images from the clinical image atlas, containing high-resolution VR-SIM images of skeletal muscle fibers, nerves with ganglia, neurovascular bundles, smooth muscle bundles in adjacent fibromuscular stroma, adipose tissue, and benign and malignant prostatic glands, along with corresponding H&E section images. Overall, there is excellent correspondence between structural features observed in VR-SIM compared to H&E. Individual nuclei are clearly resolved in the images, demonstrating the high contrast and subcellular resolution of VR-SIM.

Comparison of the full surface images of Case 6 in Figs 2 and 3, to those in the clinical atlas of Fig. 4, aids in interpretation of the features found on the VR-SIM images. Prominent features on the normal prostate surface include large areas of fascia/fibromuscular stroma; periprostatic adipose tissue admixed with vessels; neurovascular bundles; and abundant nerve fibers with wavy, elongated nuclei, including nerve ganglia. In addition in this case, on the right posterolateral surface, close to the base, a small cluster of cells with rounded nuclei was identified as a single benign prostate gland (Fig. 3d).

### Identification of path-confirmed residual cancer at the surgical margin by VR-SIM

Intra-operative assessment of VR-SIM images of RP specimens involves the identification of exposed adenocarcinoma glands on the surface of the RP specimens. To determine whether full surface VR-SIM imaging reveals suspected areas of malignant glands, two board-certified pathologists experienced in the diagnosis of prostate cancer using VR-SIM participated in consensus review of the surface images obtained in this study, with their findings compared to standard post-operative permanent histopathology of the specimens. Two cases in particular, Case 14 and Case 22, were noted as having extensive PSMs on permanent histopathology. Review of the VR-SIM images from these cases indicated that the VR-SIM findings were consistent with the histopathology findings.

**Figure 5:**
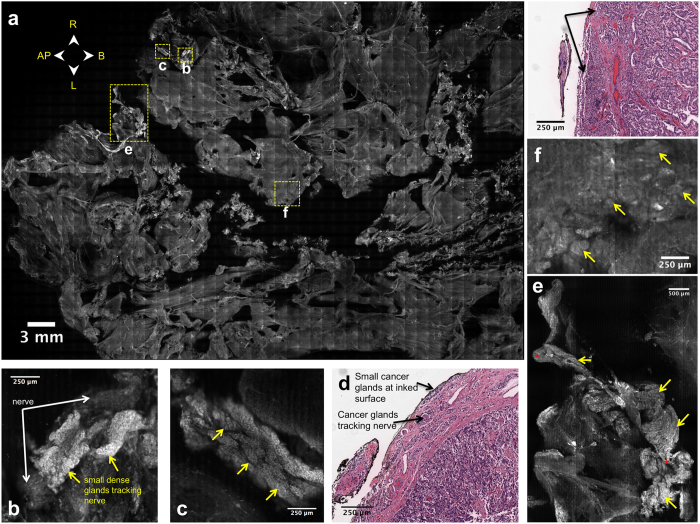
VR-SIM image of the posterior surface of Case 14, with a pathologically-confirmed positive surgical margin with perineural invasion. (**a**) Full surface image of the posterior side, with orientation provided by the directional arrows. Areas of interest on the right and central posterior surface are indicated by the dashed yellow boxes. (**b,c**) Areas of perineural invasion observed on the VR-SIM image. Small dense cancer glands (yellow arrows) are observed tracking along a prominent nerve (white arrows). (**d**) Histological cross-section from the right posterior quadrant, indicating the presence of malignant glands touching the inked surgical margin, as well as the presence of perineural invasion (glands wrapping around a nerve). (**e**) Large area of perineural invasion observed on VR-SIM, with small, dense, highly cellular glands (yellow arrows) tracking along nerve fibers (red asterisks). (**f**) Small, rounded and elliptical glands with malignant appearance (yellow arrows) on the VR-SIM image and (**g**) histological section of malignant glands at the inked surgical margin (black arrows), but not associated with nerve tissue.

Specifically, the VR-SIM image of the posterior of the Case 14 prostate (Fig. 5a) was characterized by multiple areas of small dense glands corresponding to adenocarcinoma, in some cases tracking along and intermingling with nerves at the prostate surface (Fig. 5b,c,e and Supplementary Video 2). This is consistent with the post-operative histopathology findings, where 5 foci of cancer touching the inked surgical margin in the left posterior quadrant were found. A histological section of one of these areas is shown in Fig. 5d, where small cancer glands embedded within the prostate capsule and touching the inked surgical margin are apparent. Importantly, perineural invasion (i.e., cancer glands associated with neural tissue) was observed, which is consistent with the findings of cancer glands tracking nerves on the VR-SIM surface image. One area on the VR-SIM image contained dense disorganized glands that did not appear to involve nerve tissue (Fig. 5f), which was also consistent with another histological section from that area shown in Fig. 5g, depicting cancer cells and glands touching the inked surgical margin. Post-operative pathology identified 5 foci of cancer involving the surgical margin in the left posterior quadrant, with linear extents ranging from 1 to 6 mm. Likewise, review of the VR-SIM images revealed 4 foci on the posterior surface with linear extent ranging from 3 to 8 mm, and 3 foci on the left lateral surface with linear extent ranging from 2 to 15 mm, with the largest focus covering an area of 77 mm^2^ (Supplementary Fig. S2). Although both post-operative pathology and VR-SIM imaging identified extensive cancer involvement at the margin, VR-SIM imaging revealed a larger linear extent and enabled area assessments, suggesting that surface imaging with VR-SIM could represent a more accurate assessment of extent of cancer at the surface that is not possible with the uni-dimensional sectioning approach of histopathology. Furthermore, an additional 9 mm long focus of cancer gland involvement at the surgical margin on the right lateral surface of the prostate was found with VR-SIM, suggesting an area of cancer involvement that was not identified by post-operative histopathology.

**Figure 6:**
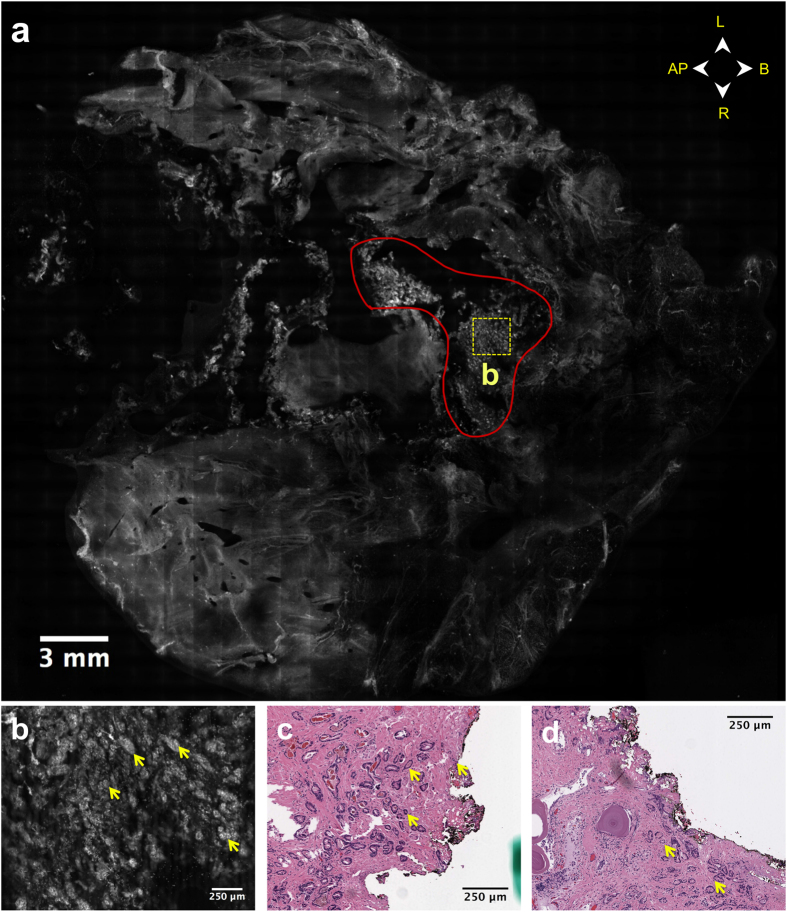
VR-SIM image of the anterior surface of Case 22, depicting a positive surgical margin due to an intra-prostatic incision. (**a**) Full VR-SIM image of the anterior surface with orientation provided by directional arrows. An area corresponding to exposed malignant glandular parenchyma is outlined in red. (**b**) Zoom of the area bounded by the dashed yellow box in (**a**). The area is characterized by extensive malignant glandular parenchyma, similar to that observed in the prostate cancer biopsy image from the atlas in Fig. 4o–p. (**c,d**) Histological confirmation of malignant glands similar to those in the VR-SIM image (yellow arrows) at the inked surgical margin, where the margin has breached the prostate capsule.

Whereas Case 14 involved primarily the presence of cancer glands invading the prostate capsule along nerves located at the surgical margin, Case 22 involved areas of exposed malignant glands in the prostate parenchyma (organ-confined) but located at the inked surgical margin. The pathology report noted the presence of two areas in the left anterior quadrant (LAQ) and left posterior quadrant (LPQ) where the surgical margin extended through the prostate capsule into the parenchyma, resulting in the presence of malignant glands at the inked surgical margin. Histologic sections of these areas are shown in Fig. 6c,d, where cancer glands are observed touching the inked surgical margin and the prostate capsule is breached, which is evidence of an intra-prostatic (capsular) incision. These findings were consistent with the VR-SIM image for this case (Fig. 6a, Supplementary Video 3). The area of small dense glands at the anterior prostate surface outlined in red in Fig. 6a and shown in detail in Fig. 6b is very reminiscent of malignant glands observed in prostate core biopsies of malignant glandular parenchyma (see for example Fig. 4o,q).

### Identification of cancer at the surgical margin by VR-SIM undetected by histopathology

**Figure 7:**
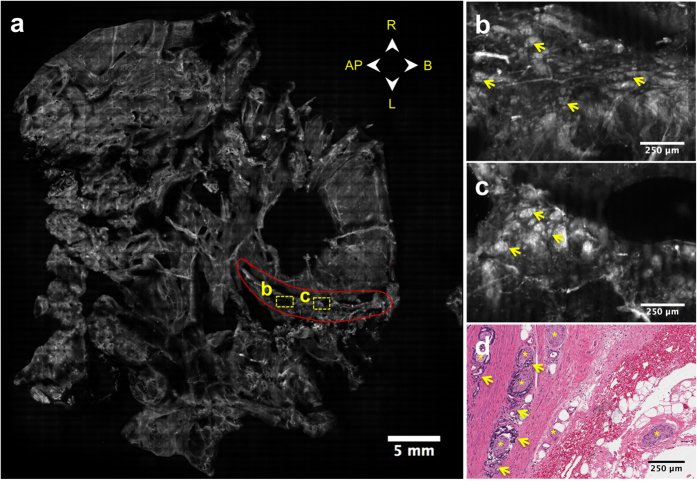
VR-SIM image of the posterior surface of Case 16 with a positive surgical margin missed by permanent histopathology. (**a**) Full surface image of the posterior surface with orientation provided by the directional arrows. A nerve with infiltrating malignant glands is outlined in red in the left posterior area, with two regions of interest bounded by yellow dashed boxes. (**b,c**) Zooms of the VR-SIM image corresponding to the dashed yellow boxes in (**a**), showing the presence of small glands infiltrating the nerve structure (yellow arrows). (**d**) Histological section from the left posterior quadrant, showing a region of malignant glands (yellow arrows) invading nerves (yellow asterisks) ~500 μm below the surgical margin, consistent with the VR-SIM findings. Although the tumor does not touch the inked margin (black arrow) in this particular section, a prominent nerve is observed at the inked margin that may be associated with the perineural invasion observed in the VR-SIM image.

Interestingly, an extensive area of glands identified as adenocarcinoma was discovered on the surgical margin in the VR-SIM image of Case 16, but the permanent histopathology report included no findings of adenocarcinoma at the inked surgical margin. Figure 7a contains the VR-SIM image of the posterior surface of the prostate from Case 16, a patient with Gleason 3 + 4 disease and a pathologic stage of pT3a with focal extraprostatic extension in the left posterior quadrant but with negative surgical margins on histopathology review. A 16 mm-long area corresponding to a large nerve tracked by and embedded with small bright glands was found in the VR-SIM image at the left posterior aspect (zoom shown in Fig. 7b,c), consistent with perineural invasion by adenocarcinoma at the surface. Although this specimen was classified as having negative surgical margins by pathology, the VR-SIM findings motivated a re-assessment of the slides from the case. Interestingly, review of the histology slides from the left posterior quadrant revealed an area of extensive adenocarcinoma and perineural invasion approximately 500 μm beneath the inked surgical margin (Fig. 7d). Although the cancer glands did not touch the inked margin in the histology slides, the fact that they were close to the margin and are consistent morphologically with the VR-SIM images of the left posterior quadrant, suggests that in this case the superior sampling coverage of VR-SIM enabled detection of a significant area of cancer involvement at the surgical margin that was missed by permanent histopathology.

### Comparison of VR-SIM and permanent histopathology findings

Table 1 contains a summary of the imaged area, image size in gigapixels, and findings of benign glands and positive surgical margins on VR-SIM imaging for each of the 19 cases analyzed in this study. In addition, the location and size of PSMs found on post-operative histopathology are included for comparison with the VR-SIM imaging results. The average total VR-SIM image area per prostate was 47.3 cm^2^ with a range of 12.6–64.8 cm^2^, corresponding to an average of 11.73 billion pixels of image data per prostate with a range of 3.1–16.1 gigapixels. Positive surgical margins were found in 8 VR-SIM prostate circumference surface images from 4 patients in this study. In addition, benign glands were observed in 8 margin surfaces from 7 patients.

In this work, VR-SIM was used to image the posterior, anterior, right, and left circumferential surfaces of the excised prostates. These imaging views overlap with the left and right posterior quadrants and left and right anterior quadrants sampled by histopathology. However, in this study we did not image the prostate base and apex in all cases. Restricting our analysis to the prostate circumference areas imaged by VR-SIM, and excluding those specimens that were positive for cancer at the apical margin only, then VR-SIM imaging was able to detect cancer involvement at the surface in 3 of 4 patients with path-confirmed positive surgical margins, and 3 of 3 with PSMs with a linear extent greater than 500 μm. The one case with a circumferential PSM that was missed by VR-SIM was Case 6, which had a <500 μm area of cauterized tumor at the left posterior margin. Although a 1-mm long feature on the left posterior VR-SIM image (Supplementary Fig. S3) was identified as malignant by one reviewer, the pathology reviewers could not reach agreement on this; therefore it was ultimately eliminated from further consideration. The remaining 3 path-confirmed PSM cases, all of which contained confirmed areas of margin involvement of at least 1 mm in extent, were detected by VR-SIM imaging. In addition, VR-SIM identified a 16-mm PSM on the posterior aspect of Case 16, although post-operative histopathology found no PSM at any surface in this patient.

## Discussion

Although there has been much progress in surgical techniques for radical prostatectomy, achieving the opposing goals of complete radical resection for oncologic safety, and limited resection for maximal preservation of prostate-adjacent neurovascular bundles, is a challenge for even the most experienced urologic surgeons. Consequently, the incidence and negative consequences of positive surgical margins continues to be a critical issue that has not been alleviated by the use of limited-sampling intra-operative pathology techniques. As a potential alternative, this report demonstrates the first circumferential large-area microscopic imaging of radical prostatectomy surfaces with histologic resolution and contrast, collected using fluorescence structured illumination microscopy. Importantly, we prove that it is feasible to image nearly the entire prostate organ surface in timeframes amenable to intra-operative use. Specifically, after an initial optimization phase, we consistently imaged at least 4 anatomical surfaces of the prostate in 18 patients at 1.3 μm lateral image resolution in one hour or less. Since the overall imaging throughput is currently limited by the speed of the scanning microscope stage and not by the image acquisition speed, further engineering refinements to speed up the scan stage movements make it possible to reduce this imaging time to 20 minutes or less, which is well within desirable intra-operative time constraints compared to frozen section analysis.

VR-SIM captured and identified the locations of malignant prostatic glands at the prostate circumferential surgical margin in 3 of 4 radical prostatectomy specimens, and identified all areas of residual cancer at the surgical margin of 1 mm extent or greater. Furthermore, VR-SIM identified residual cancer at the surgical margin in an additional patient that was classified as negative for margin involvement by permanent histopathology. The ability to image the prostate surface directly raises a tantalizing possibility, namely that the utility of this technology could go beyond the operating room into the pathology suite. Specifically, VR-SIM covers vastly more surface area than permanent histopathology, thereby enabling, *inter alia*, an assessment of the *area* of cancer involvement at the margin as opposed to just linear extent. Post-operative 5-year recurrence rates have been observed to be directly correlated with extent of tumor at the surgical margin, with higher recurrence rates associated with more extensive tumor involvement at the margin^36^. However, the limited sampling approach of permanent histopathology may compromise the ability to accurately characterize the tumor extent at the surgical margin. The use of VR-SIM in the pathology lab to more accurately assess tumor extent at the margin could improve the precision with which clinicians are able to differentiate the risk of recurrence and prescribe adjuvant treatment.

Taken together, these results indicate the potential clinical utility of VR-SIM imaging. The use of this technology was able to identify prostate cancer invading into and beyond the prostate capsule to the surgical margin, as well as surgically-exposed organ-confined cancer. Since the definition of a PSM in prostate cancer is “tumor extending to the surface of the prostate wherein the surgeon has cut across the tissue plane,”^36^ the shallow imaging depth of VR-SIM is sufficient to detect PSMs. Had VR-SIM been used in the operating room, actionable information about the presence and extent of residual cancer at the surgical margin could have been provided to the surgeons in a timely manner, possibly preventing uncorrected PSMs in 4 patients. Importantly, had VR-SIM been used either in the operating room *or* the pathology suite, it would have identified a PSM missed by permanent histopathology that would have implications for adjuvant treatment decisions. Direct VR-SIM imaging of the entire prostate surface thus provides a major advantage for intra-operative PSM detection over histopathology methods that involve cutting the specimen, since it can be performed over a more comprehensive circumferential area, near the patient in a shorter timeframe, and does not compromise downstream permanent pathology processing procedures. The generated images are sufficiently detailed to clearly identify individual cell nuclei and glandular morphology which allows for pathologic diagnosis^33^. Also, because VR-SIM is applied to the intact circumferential tissue surface instead of the standard protocol of cross-sections of the organ, the actual tissue area that needs to be examined by the pathologist to assess margin status is much less than in the standard protocol, since most of the tissue area in cross-sectional H&E slices is away from the surgical margin of interest. In fact, the mean VR-SIM image area in fully imaged prostates was 49 ± 10 cm^2^, whereas the mean total area of H&E-stained tissue mounted on slides for these cases was 138 ± 22 cm^2^. Thus, although the VR-SIM images obtained of the prostate circumference are large, they actually represent a three-fold reduction in tissue area that must be examined by the pathologist to assess margin status specifically than in standard H&E, because of the difference in how the tissue is sampled.

A limitation of the current study was that the prostate base and apex were not routinely imaged with VR-SIM, and 4 patients had positive surgical margins at the apex only, which precluded a direct comparison between VR-SIM and histopathology in these cases. PSMs at the prostate base were not observed in this study and are generally uncommon^36^, so the significance of not imaging them with VR-SIM is likely low. However, consistent with other reports on location of PSMs in robot-assisted laparoscopic prostatectomy, the most common site of a PSM in this study was the apex^37^. The prognostic significance of apical PSMs is the subject of some debate, whereas some studies have shown that the presence of an apical PSM does not independently predict biochemical recurrence (i.e., elevation of post-operative prostate specific antigen (PSA) which is the first indicator of cancer recurrence)^38^. On the other hand, the prognostic significance of posterolateral (circumferential) PSM is more clear, being associated with an elevated risk of biochemical recurrence^39^. The apex was not imaged in this initial patient series because the prostate does not independently stand vertically on the conical apex surface. However, since realizing the frequency of apical margin involvement we have devised a method to image the apex by positioning the prostate vertically in a cylindrical apparatus, which maintains stable contact between the apical surface and the imaging slide and enables imaging of the full apical surface (Supplementary Fig. S4). This method is being employed in the continuation of this study. An additional limitation is related to the presence of microscopic liquid droplets, which appear as tiny “holes” in the image, as well as areas where the tissue height varies, compromising perfect contact with the imaging slide. We have developed rapid autofocus strategies^40^ which could be leveraged in future studies to overcome variations in tissue topography, once the stage movement time is reduced to compensate for additional time needed to achieve focus at each frame. At any rate, even with the current protocol the surface coverage of SIM represents a vast improvement over sparse sectioning protocols in terms of area sampling.

Positive surgical margins, whether associated with surgical incision of an organ-confined tumor, or associated with extraprostatic extension of the tumor found at the edge of the excised specimen, are widely accepted as a poor prognostic indicator and should be avoided. Patients with positive surgical margins must undergo intensive post-operative PSA screening to screen for cancer recurrence, and often, adjuvant salvage radiotherapy is performed, especially for higher risk diseases. Although additional surgery is not performed, the use of adjuvant radiotherapy is harmful, and can undo the positive benefits of nerve sparing surgeries and results in further co-morbidities. Currently available intra-operative pathology techniques have failed to eliminate the widespread occurrence of incomplete tumor resections, in part because of severe sampling limitations caused by the need to physically section the specimen. In this work, we address this limitation with a rapid optical sectioning scanner, optimized for intra-operative use and enabling direct imaging of the uncut tissue surface. Compared to emerging intensive FSA techniques such as NeuroSAFE^41^, or other competing imaging technologies, VR-SIM has a number of advantages that could promote its eventual widespread adoption in the surgical suite. The use of high-speed optical sectioning is non-destructive and enables high-resolution and high-contrast imaging of the true prostate circumference, covering most of the prostate surface in a timeframe on par with that of emerging intensive FSA techniques^41^. The images can be collected by a single technician; thus personnel requirements are low compared to emerging intensive FSA techniques^41^, making it more likely to be adopted at non-academic medical centers, ambulatory or private surgery centers, and community hospitals where many radical prostatectomy procedures are performed. Finally, the images produced by SIM are inherently digital thereby facilitating telepathology, and are very similar to those of traditional histopathology, with only minimal adjustment needed by the pathologist to examine the grayscale images on the basis of structure and brightness rather than structure and color. This technology has the potential to impact clinical workflow by enabling additional surgical resection during the initial operation, similar to Mohs surgery for skin cancer. The presence and location of a PSM would be communicated to the surgeon, who would then excise additional tissue from the cavity in the area of concern. The availability of this technique could lead to changes in clinical practice, by enabling surgeons to perform more nerve-sparing surgeries with VR-SIM serving as an intra-operative safety net, and by significantly reducing the need for harmful post-operative therapies. Although extensive validation of the technique for detecting clinically-significant residual tumor in a larger patient series will be important, these results position VR-SIM as a technology that could replace frozen section analysis in the operating room, ultimately eliminating incomplete cancer resections and the associated negative health outcomes.

## Materials and Methods

### Study design

The objective of this study was to evaluate the feasibility and clinical potential of circumferential imaging of whole human prostates using VR-SIM for timely detection of residual cancer at the surgical margin. The study consisted of two primary experiments. The first experiment, conducted on a fresh cadaveric prostate obtained under a protocol approved by the Tulane University Biomedical Institutional Review Board, was designed to augment a clinical atlas of VR-SIM images of prostate surface structures against the corresponding *en face* H&E histopathology section. The second experiment was conducted on fully-intact radical prostatectomy specimens obtained immediately after surgery, and was designed to optimize the imaging protocol and to determine the feasibility and clinical potential of VR-SIM for intra-operative circumferential prostate microscopic imaging. Under a protocol approved by the Tulane University Biomedical Institutional Review Board, men undergoing radical prostatectomy (n = 24) for confirmed prostate cancer provided informed consent to participate in this experiment. The radical prostatectomy specimens were obtained immediately after surgery and retained for imaging studies for approximately one hour before being submitted to the Tulane University Hospital Pathology Department for standard-of-care processing. The RP specimens were not cut in any manner prior to imaging, which was performed directly on the prostate surface. The surgeons (co-authors BRL, MMM, and WL) were blinded to the imaging results and there were no changes to the patient standard-of-care. All protocols were approved by the Tulane University Biomedical Institutional Review Board, and all methods were carried out in accordance with the approved guidelines.

### Instrumentation

We previously reported the development of the VR-SIM system^32^ and validation on large prostate core biopsies^33^. Briefly, the system is constructed around an automated epi-fluorescence microscope platform (RAMM, Applied Scientific Instrumentation), which incorporates a 7 mm/s motorized XY specimen stage and a motorized Z objective positioner. Blue excitation light is provided by an LED (475 nm, Thorlabs). Excitation light is transmitted through a polarizing beam splitter and imaged onto a liquid crystal on silicon (LCoS) spatial light modulator (SLM, Model 3DM, Forth Dimension Displays). The excitation light is filtered and reflected into the imaging objective (Nikon, Plan Apo 10 × 0.45 NA) by a 500 nm edge dichroic mirror (FITC-Di01-Clin-25 × 36, Semrock), projecting the SLM-generated pattern onto the sample. Fluorescence from the sample is collected by the objective and transmitted through the dichroic mirror and a 515 nm longpass emission filter (FITC-LP01-Clin-25, Semrock). The image is collected by a scientific CMOS camera (Orca Flash 4.0 v2, Hamamatsu) at a full-frame resolution of 2048 × 2048 pixels with a pixel size of 6.5 μm. Thus, at 10X magnification, the single-frame field-of-view is 1.3 mm × 1.3 mm and the lateral resolution of the system is 1.3 μm, in this case limited by the Nyquist criterion (0.65 μm/pixel at the sample).

Synchronization and control of the LED, SLM, stage, objective, and camera is achieved *via* custom-written LabVIEW software (National Instruments) and home-built electronic triggering circuits. The VR-SIM module is mounted to the RAMM base and fits comfortably on a 24″ × 36″ passive isolation breadboard (Nexus, Thorlabs) mounted on a 3′ × 4′ wheeled lab bench (OnePointe Solutions) (Fig. 1a). Incoherent SIM is performed by projecting a sinusoidal pattern onto the sample, which is phase-shifted by one-third of the grid period between each of three sequential images. From these three patterned images, a single optically-sectioned image is obtained using the square-law detection algorithm described by Neil *et al.*^34^:


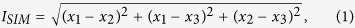


where *I*_*SIM*_ is the recovered optically sectioned image, and *x*_1,_ *x*_2_, and *x*_3_ are the three sequential patterned images, respectively.

### VR-SIM image mosaic processing

Mosaics of the stained tissue were collected using a serpentine scan approach. Each individual frame in the mosaic was first intensity-normalized, and then corrected for non-uniform illumination (i.e. flat field correction) by dividing by an intensity-normalized reference image taken of a fluorescent calibration slide (Chroma). To maximize the area-throughput, there was no overlap between adjacent images during collection, and mosaics were constructed without the use of stitching algorithms. The processed images were re-scaled to restore them to 16-bit grayscale intensity and saved as full resolution TIFF or BigTIFF format. The images were then subsequently converted to multi-resolution tiled pyramidal TIFF or BigTIFF format using nip2 software. Multi-resolution format images were uploaded to a web-based image viewer with zoom-and-pan capability for remote review by the pathologists.

### Cadaveric prostate imaging and pathologist review

In order to simulate a capsular incision of the prostate, we obtained a portion of a benign prostate (surface measuring approximately 2 cm × 3 cm) from a male cadaver under a Tulane University Biomedical Institutional Review Board-approved protocol. The specimen was transported to the imaging lab and immediately rinsed with PBS to remove excess blood or fluid from the surface. The prostate was then blotted dry with lab tissue and 0.1% acridine orange in phosphate buffered saline (PBS) was sprayed on the surface and allowed to remain for 30 seconds. Following the staining, the prostate was rinsed in PBS and again blotted dry. The intact prostate surface was imaged with VR-SIM; then, a capsular incision was created by shaving an approximately 3 mm-thick section from a portion of the intact prostate specimen surface. The new tissue surface was stained and imaged again, then marked with histological inks and placed in formalin for histological processing, after which a single *en face* section of the marked surface was obtained for H&E staining and correlation with VR-SIM. The H&E section was digitized at 200X (20X objective) magnification with an Aperio whole slide scanner (Leica Biosystems). The VR-SIM image and the H&E section image were reviewed to identify corresponding structures, which were included in the clinical image atlas, along with benign and malignant prostate biopsy images collected in a prior study^33^.

### Radical prostatectomy imaging and pathologist review

Intact prostates obtained from robotic radical prostatectomy procedures were brought directly from the operating room to the nearby imaging lab within 10 minutes of excision. The specimen was immediately rinsed with PBS to remove excess blood or fluid from the surface. The prostate was then blotted dry with lab tissue and sprayed with 0.1% acridine orange in phosphate buffered saline (PBS) which was allowed to remain wet on the surface for 30 seconds. Following the staining, the prostate was rinsed in a beaker containing PBS and again blotted dry. Specimens were then directly imaged with VR-SIM as described previously without any cutting of the specimen. Specimens from the first 5 patients were used to optimize the imaging parameters of the VR-SIM system. Rather than attempting to image all surfaces of the prostate in the first set of patients, the objective for these cases was to finalize imaging settings to be used for all future specimens. We investigated different pattern spatial frequencies for SIM imaging to determine which gave the best image quality. Specifically, we tested two normalized pattern spatial frequencies (*v* = 0.036 and *v* = 0.018), where *v* = *f*λ/NA, *f* = absolute pattern frequency at the sample, λ = wavelength, and NA = numerical aperture of the objective lens. In our previous work we found that these normalized spatial frequencies in our system resulted in measured optical section thicknesses of 24 μm and 45 μm, respectively, defined in this case as the half-width-half-maximum (HWHM) of the axial response^32^. In the current specimens we found that a normalized pattern frequency *v* = 0.018 resulted in the best balance between background rejection/contrast/effective resolution and signal-to-noise ratio (SNR). To minimize image acquisition time while maintaining high image quality, we also investigated the use of different camera integration times and LED intensities. Based on the results of this initial optimization phase, the integration time used for all subsequent specimens was kept constant at 10 ms (30 ms per 2048 × 2048 pixel SIM frame) and the LED was set to its highest current setting. A 5 ms-duration bitplane sequence was used on the SLM. Altogether, these settings were found to result in a satisfactory signal level and image contrast, while minimizing the effect of integration time on the total image acquisition time.

The next set of 19 patients was used to test clinical feasibility and potential utility, by focusing on full circumferential surface imaging in <1 hour and identification of pathologically relevant features on the surfaces from the resulting images. For each specimen, the prostate surface was placed on a 7.5 × 5 × 0.1 cm glass slide and gentle pressure was applied so that the surface of the prostate adhered to the glass (Fig. 1c). The slide was then mounted on the stage of the VR-SIM system; no compression was applied to the prostate during imaging. After completely imaging one entire surface with VR-SIM, the specimen was manually rotated along the urethral axis (Fig. 1c) and the imaging procedure was repeated until all possible surfaces of the prostate (i.e. posterior, anterior, right lateral, or left lateral) were imaged within a one-hour timeframe (Fig. 1d,g).

### Permanent histopathology processing and pathologist review

At the conclusion of imaging, radical prostatectomy specimens were submitted to histology to undergo standard-of-care processing. Specifically, the external capsular tissue margin was appropriately inked for surgical margin evaluation. The apex was removed and radially sectioned. The remainder of the prostate was then quartered into gross quadrants consisting of the left anterior, right anterior, left posterior, and right posterior. Each quadrant was serially sectioned from apex to base at intervals of approximately 3 mm and each slice was subsequently placed in cassettes for formalin fixation for 24 hours, followed by paraffin embedding. From each 3 mm-thick slice a single 4 μm section was cut and mounted on a microscope slide, stained with standard H&E, and reviewed by a pathologist in order to determine the margin status, as is consistent with standard of care at our institution (Supplementary Fig. S1). This process resulted in an average of 51 ± 15 individual microscope slides for pathologist review for the specimens analyzed in this study. From the post-operative pathology report and further consultation with a co-author pathologist (ABS), we classified positive surgical margins as the presence of tumor cells or glands (including cauterized cells) at the inked edge of the specimen, according to the standard of care at our institution. Images of relevant H&E-stained tissue sections were scanned using the Aperio slide scanner.

In a majority of procedures we completed the analysis and forwarded the fresh tissue to histology within the 1 hour time frame. Once returned, the tissue was immediately sectioned and placed in formalin to maximize exposed tissue surface area and hence, formalin fixation. To date, no histologic artifacts or untoward tissue autolysis has been noted during standard of care downstream histologic analysis at our institution.

### VR-SIM image pathologist review

Two board certified pathologist co-authors (ABS and HZK), and non-pathologist co-authors trained in viewing VR-SIM images reviewed all images obtained in the study for regions of interest (ROIs) containing suspected prostatic glands at the surgical margin. The assembled ROIs were then reviewed individually by the two pathologists using VR-SIM images of confirmed benign and malignant prostate glands from the clinical imaging atlas as a reference, and were assigned a rank of 1 to 5, where 1 indicated most likely to be benign, 2 indicated possibly benign, 3 indicated equivocal between cancerous or benign, 4 indicated possibly cancerous, and 5 indicated most likely to be malignant. ROIs with rankings of 1–2 were designated as benign, and ROIs with rankings of 4–5 were designated as malignant. Following this initial review, ROIs in which the rankings of the two pathologists were disparate (i.e. one pathologist ranked benign and the other ranked malignant) were re-reviewed and discussed by the two pathologists to achieve a consensus ranking for the ROI. Consensus was achieved in all ROIs except for a 1-mm ROI from the left lateral surface of Case 6 (Supplementary Fig. S3), which was eliminated from further consideration. The maximum linear extent and area of malignant ROIs were measured using FIJI. In addition to the VR-SIM image review, the pathologists reviewed the postoperative pathology reports from the radical prostatectomy cases, as well as the corresponding H&E slides, and compared the clinical findings to the observations from the VR-SIM images.

### Data and materials availability

De-identified VR-SIM and H&E images are available for use in accordance with provision of informed consent under Tulane University Biomedical IRB project #623770-3. To obtain data contact J.Q.B.

## Additional Information

**How to cite this article**: Wang, M. *et al.* Gigapixel surface imaging of radical prostatectomy specimens for comprehensive detection of cancer-positive surgical margins using structured illumination microscopy. *Sci. Rep.* **6**, 27419; doi: 10.1038/srep27419 (2016).

## Supplementary Material

Supplementary Information

Supplementary Video 1

Supplementary Video 2

Supplementary Video 3

## Figures and Tables

**Table 1:** Comparison of VR-SIM consensus pathology results and permanent histopathology findings of positive surgical margins, by location.

Case ID	Area (cm^2^)	Gigapixels	VR-SIM Consensus Pathology Status (by location)						Location, size of PSM on pathology
			Posterior	Anterior	Right	Left	Base	Apex	
6	60.5	15.0	–	–	Benign glands	–	NI	NI	Left Posterior, <0.5 mm
7	58.7	14.6	–	Benign glands	–	–	NI	NI	–
8	48.0	11.9	–	–	–	–	–	NI	–
9	25.8	6.4	–	–	–	–	NI	NI	–
10	12.6	3.1	–	NI	NI	NI	NI	NI	–
11	53.1	13.2	–	Benign glands	–	–	NI	NI	–
12	48.6	12.1	Benign glands	–	–	–	NI	NI	Apex, periurethral, 2 mm
13	42.9	10.6	–	–	–	–	NI	NI	–
14	64.8	16.1	PSM, multifocal, 3–8 mm	–	PSM, 9 mm	PSM, 2–15 mm	NI	NI	Left Posterior, 5 foci, 1–6 mm
15	45.7	11.3	–	–	–	–	NI	NI	–
16	47.8	11.9	PSM, 16 mm	–	–	–	NI	NI	–
17	37.5	9.3	Benign glands	–	–	–	NI	NI	Apex, focal
18	44.3	11.0	–	–	–	–	NI	NI	–
19	38.1	9.5	–	–	–	–	NI	NI	–
20	62.0	15.4	–	Benign glands	–	–	NI	NI	Apex, focal <1 mm
21	45.3	11.2	–	Benign glands	–	Benign glands	NI	NI	–
22	56.3	14.0	PSM, 3 mm	PSM, 12 mm	–	PSM, 2 mm	NI	NI	Left Posterior (8 mm) and Left Anterior (2 mm)
23	48.1	11.9	–	–	–	–	NI	NI	Apex, focal
24	57.9	14.4	–	PSM, 1 mm	–	–	NI	NI	Right Posterior (focal) and Base (focal)

NI = Not imaged.
