# Supplementary material for: Gigapixel surface imaging of radical prostatectomy specimens for comprehensive detection of cancer-positive surgical margins using structured illumination microscopy
Source: Sci Rep. 2016 Jun 3;6:27419. doi: 10.1038/srep27419 (PMC4891779; doi:10.1038/srep27419)
Supplement: Supplementary Information [file srep27419-s1.pdf]

Supplementary Information

Gigapixel surface imaging of radical prostatectomy specimens for comprehensive detection of cancer-positive surgical margins using structured illumination microscopy

Mei Wang, David B. Tulman, Andrew B. Sholl, Hillary Z. Kimbrell, Sree H. Mandava, Katherine N. Elfer, Samuel Luethy, Michael M. Maddox, Weil Lai, Benjamin R. Lee, J. Quincy Brown

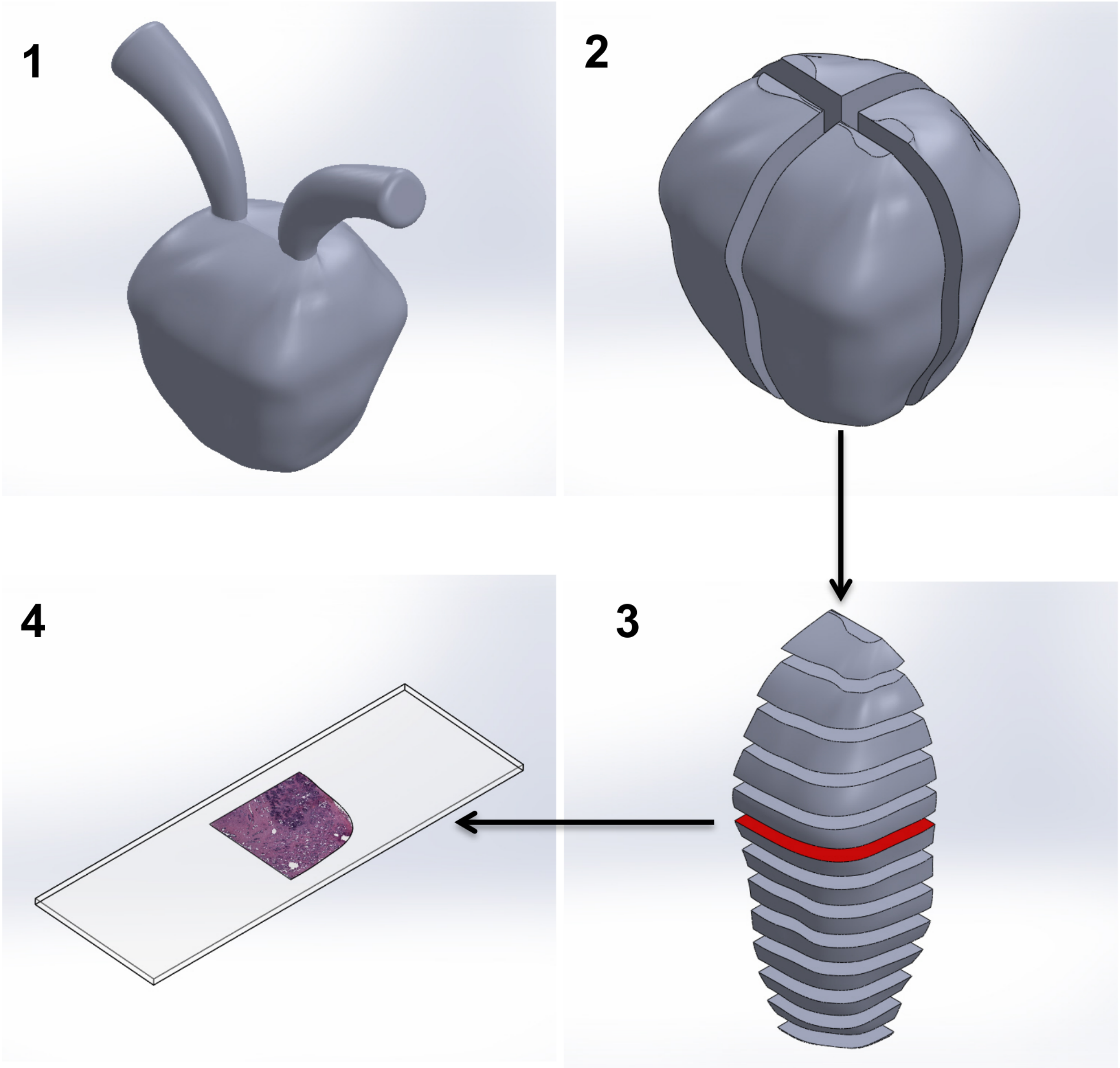

Supplementary Fig. S1. Flowchart of standard radical prostatectomy histological processing. 1) The intact prostate specimen with seminal vesicles and ductus deferens attached. VR-SIM imaging was performed directly on the surface of the specimen in this state. 2) For histological processing, the seminal vesicles and ductus deferens are first removed. Then, the prostate is quartered as shown. 3) Each quarter of the prostate is grossly sectioned into approximately 3 mm-thick cross-sections. The red surface on one of the slices indicates the 4 μm plane that is subsequently sectioned with a microtome and 4) mounted onto a slide for H&E-staining and pathologist review. In this example, histological processing would result in 52 slides, of which only a very small amount of tissue actually corresponds to the outer prostate surface.

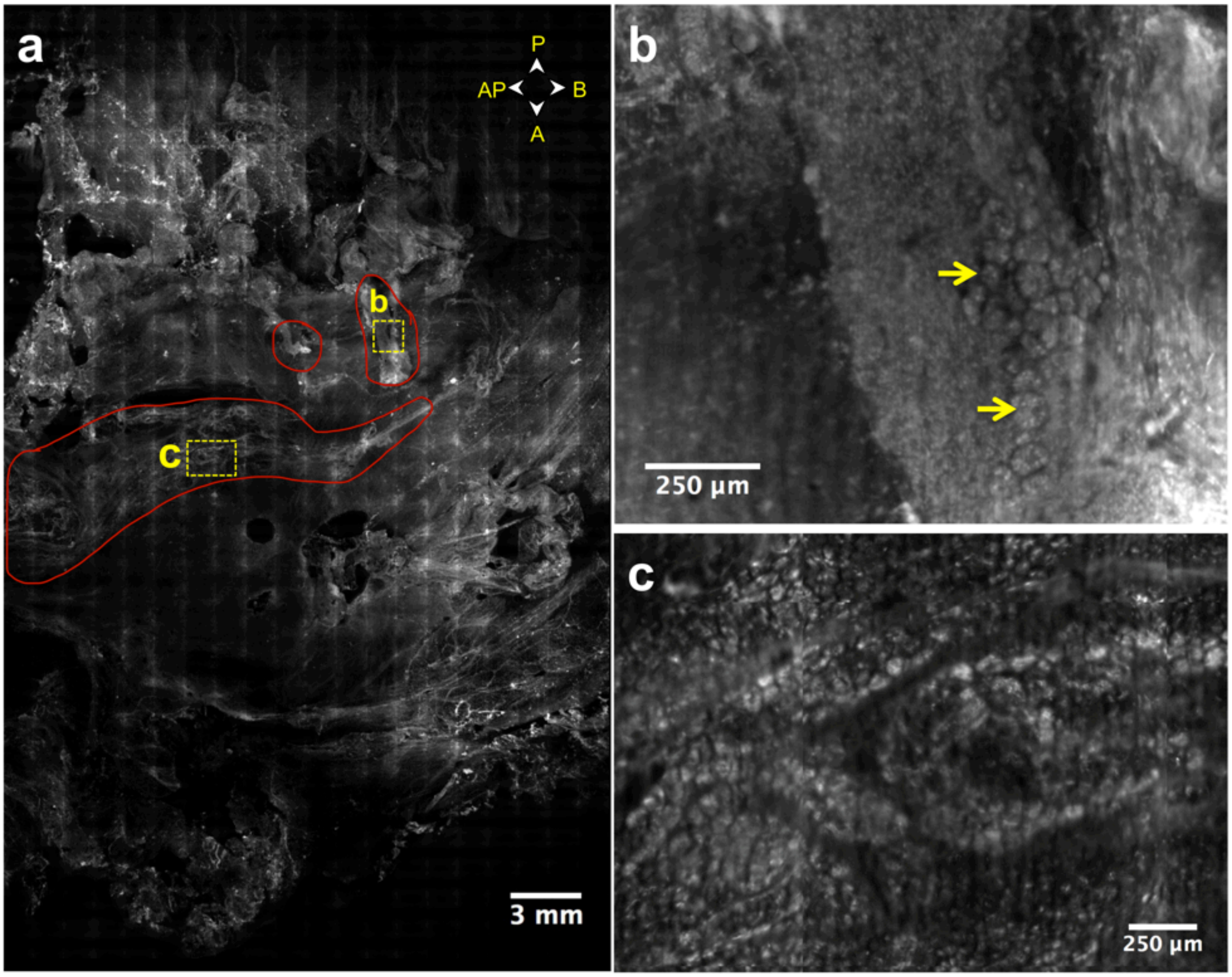

Supplementary Fig. S2. VR-SIM image of the left lateral surface of Case 14 with areas of extensive tumor involvement. a) Full surface image of the left lateral surface with orientation provided by the directional arrows. Areas of tumor involvement on the mid- to posterior-left surface are outlined in red, the largest of which was 15 mm long and occupied an area of 77 mm^2. b-c) Zooms of the yellow dashed boxed areas in (a), showing the presence of small, dense, irregular glands identified by the pathologists as cancer. Permanent histopathology identified 5 foci of tumor involvement at the inked surgical margin on the left posterior quadrant, which is consistent with the VR-SIM findings.

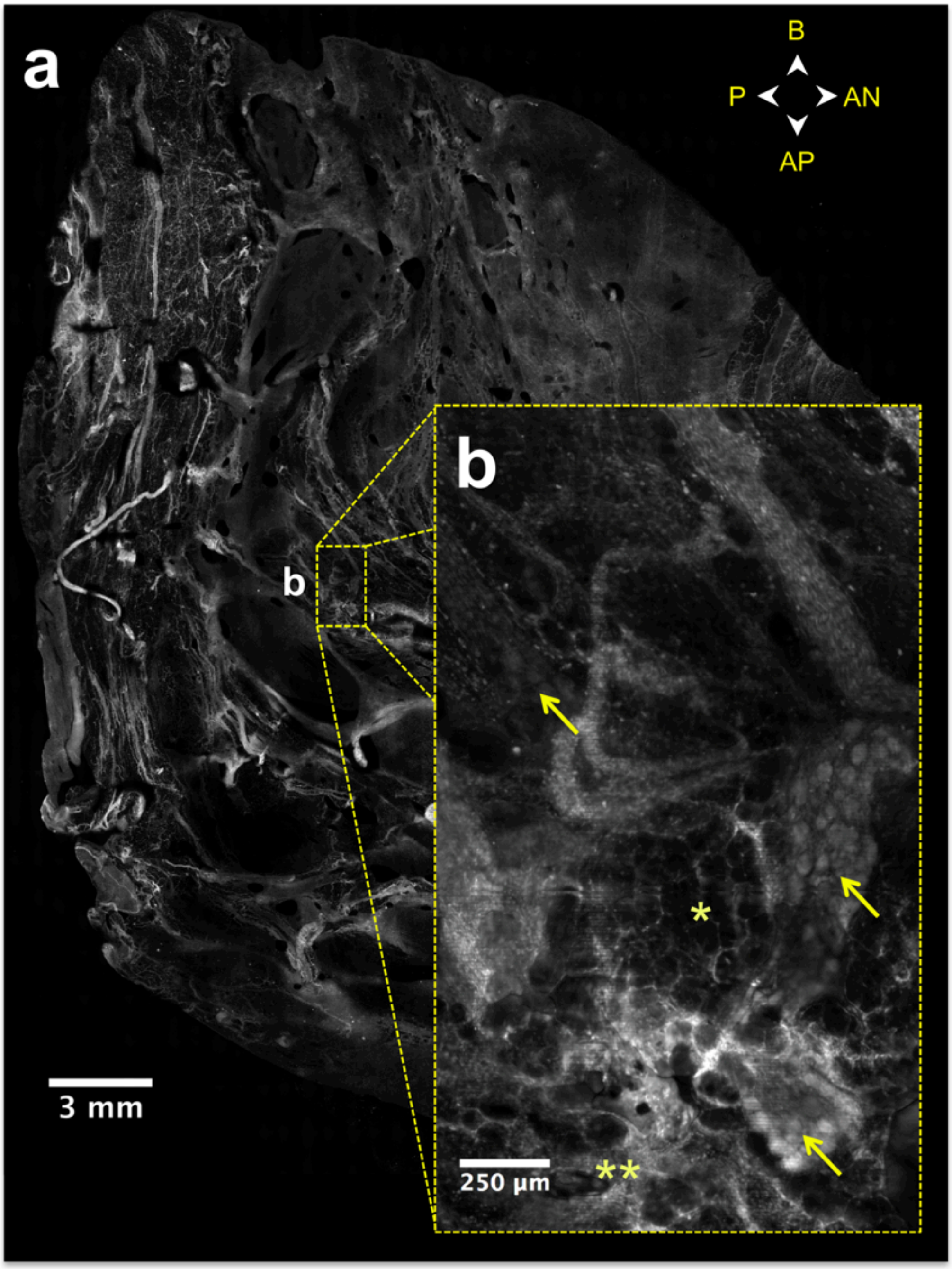

Supplementary Fig. S3. Suspicious area on left posterior surface of Case 6. Zoom of an area on the left posterior aspect of the prostate of Case 6 that was identified as suspicious. Arrows point to small irregular structures reminiscent of cancerous glands, although individual cell nuclei are not apparent as is usual for cancerous glands observed on SIM. The double asterisk (**) indicates an area of adipose tissue that was suspiciously highly cellular, compared to the fewer nuclei in the normal appearance of adipose tissue (single asterisk *). Although a small focus (<500 µm) of cauterized tumor at the surface was found in a single H&E slide on the left posterior quadrant, pathologist reviewers were divided on the classification of this area on the VR-SIM image as malignant or benign, resulting in a false negative. We expect to resolve diagnostic conundrums of this type as we continue to validate the method in a larger patient series.

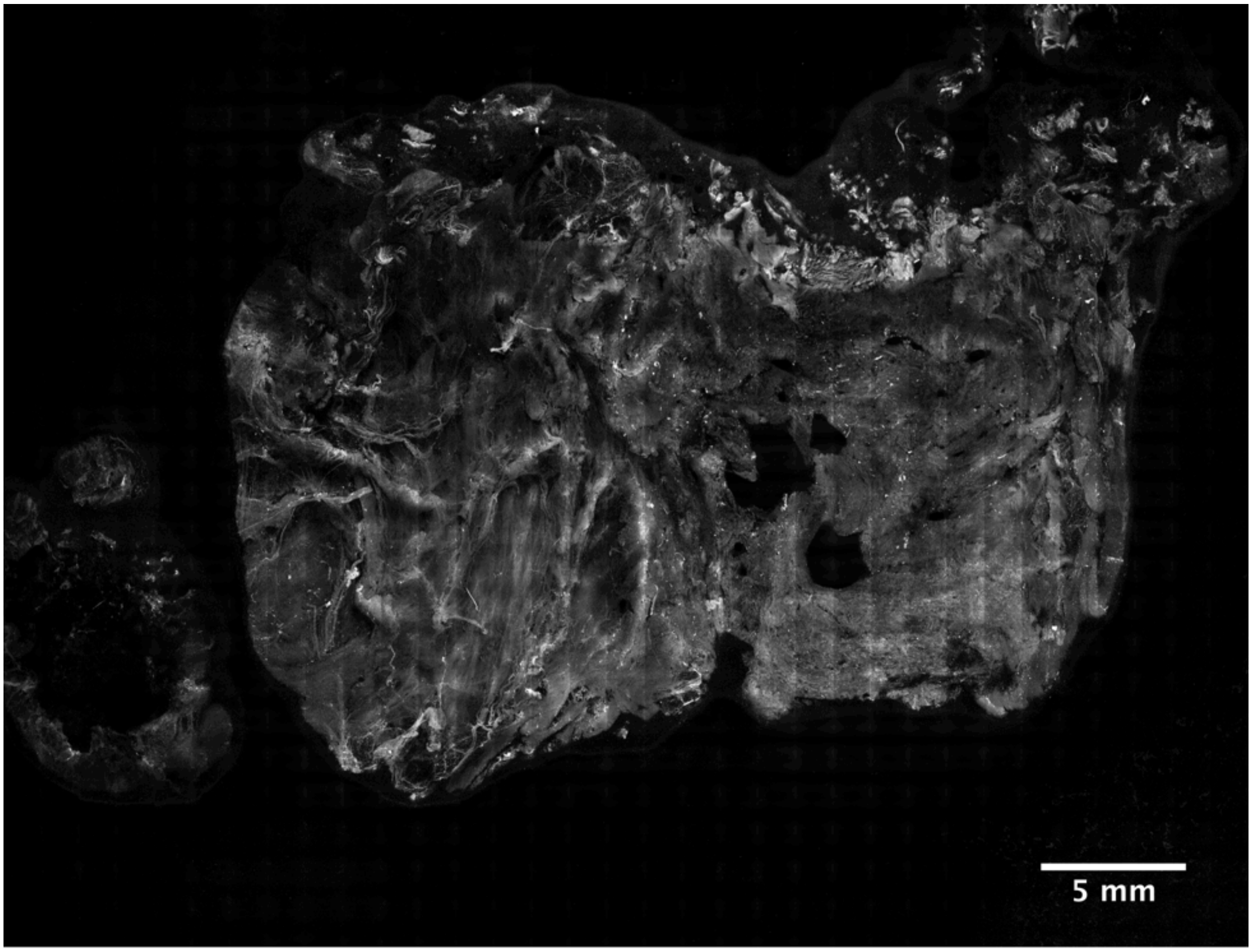

Supplementary Fig. S4. VR-SIM image of the apical prostate surface. The prostate apex was imaged by standing the prostate on the apical end, supported by a cylindrical apparatus. (Image was collected from a prostate subsequent to the patient series described in this paper).

Supplementary Table S1. Summary of tumor stage, Gleason grade, number of histology slides generated, and post-operative histology status by location. Linear extent of tumor is listed in cases in which it was noted in the pathology report. PSM = positive surgical margin. RPQ = right posterior quadrant, LPQ = left posterior quadrant, RAQ = right anterior quadrant, LAQ = left anterior quadrant). '-' implies a cancer-negative surgical margin.

				Post-operative histology status (by location)					
**Case ID**	**Stage**	**Gleason grade**	**# slides**	**RPQ**	**LPQ**	**RAQ**	**LAQ**	**Base**	**Apex**
**6**	pT2c	3+4	44	-	PSM, focal, <0.5 mm	-	-	-	-
**7**	pT2a	3+4	47	-	-	-	-	-	-
**8**	pT2c	3+4	40	-	-	-	-	-	-
**9**	pT3a	3+3	40	-	-	-	-	-	-
**10**	pT2c	3+4	43	-	-	-	-	-	-
**11**	pT3b	3+4	50	-	-	-	-	-	-
**12**	pT2c	3+4	43	-	-	-	-	-	PSM, 2mm, periurethral
**13**	pT2c	3+4	62	-	-	-	-	-	-
**14**	pT3a	4+3	102	-	PSM, multifocal, 1-6 mm	-	-	-	-
**15**	pT3a	4+3	40	-	-	-	-	-	-
**16**	pT3a	3+4	42	-	-	-	-	-	-
**17**	pT2c	3+4	57	-	-	-	-	-	PSM, focal
**18**	pT3a	4+3	33	-	-	-	-	-	-
**19**	pT2c	3+4	67	-	-	-	-	-	-
**20**	pT2c	3+4	60	-	-	-	-	-	PSM, focal, < 1 mm
**21**	pT3a	3+3	48	-	-	-	-	-	-
**22**	pT2c	3+3	49	-	PSM, 8 mm	-	PSM, 2 mm	-	-
**23**	pT2c	3+4	50	-	-	-	-	-	PSM, focal
**24**	pT3b	5+4	60	PSM, focal	-	-	-	-	-

Supplementary Video 1. Multi-scale viewing of gigapixel VR-SIM images using multi-resolution image viewer, Case 6, right lateral margin.

Supplementary Video 2. Identifying prostate cancer at the prostate surface, Case 14, posterior margin.

Supplementary Video 3. Identifying exposed malignant glandular parenchyma, Case 22, anterior margin.
